# Innovative Foods: The Future Food Supply, Nutrition and Health

**DOI:** 10.3390/foods12071359

**Published:** 2023-03-23

**Authors:** Malik Altaf Hussain, Alaa El-Din Ahmed Bekhit

**Affiliations:** 1 School of Science, Western Sydney University, Richmond, NSW 2753, Australia; 2 Department of Food Science, University of Otago, Dunedin 9054, New Zealand

## 1. Introduction

In the coming decades, feeding the growing world population is going to become a global food-supply challenge for our existing food systems. At present, the global food-supply chain has been severely affected due to disruptions caused by the COVID-19 pandemic, climate change, and political conflicts. These disruptions have led to substantial increases in food prices (e.g., FAO cereal Price Index increased by about 25 points and vegetable oil Price Index increased by more than 60 points in March 2022, https://www.statista.com/chart/20165/un-global-food-price-index/, accessed on 22 February 2023). The global food-production crisis and lack of sustenance affordability can create further regional food-security and political disruptions and trigger further socioeconomical injustice among various nations. Innovations to reshape global food systems through improving local food-production capabilities, enabling infrastructure for agricultural innovation, and facilitating knowledge flow as well as technology dissemination are necessary to curb food shortages and security. Developing and applying new and emerging technologies, including synthetic biology and artificial intelligence, to modernize food production and processing would strengthen efforts to overcome supply challenges in the future.

Currently, food-innovation and product-development activities are heavily focused on new protein sources to provide alternatives to animal-based foods, which have been negatively perceived in certain societies. While the current approaches mostly focus on producing appealing and nutritionally comparable products to those traditionally obtained from animals, some key nutritional and health aspects of the long-term consumption of these alternative foods have not yet been studied in detail. For example, the allergenicity risk to public health from emerging protein sources such as insects, microalgae, cultured meats, and legumes must be managed, as these foods are being introduced in the global food supply. The risk associated with the long-term lack of certain nutrients (minerals and vitamins found in animal products) and the presence of antinutrients (e.g., lectins and alkaloids in plants) require urgent evaluation. Novel food-production approaches are equally important for improving food-handling and transportation practices, developing processing technologies to enhance the utilization of foods, and providing maximum nutritional benefits to the consumer. Food safety and public health protection are also key elements of emerging global food systems.

## 2. Food Innovation and Food Security

Food security requires a sustainable, adequate supply of food that is accessible to the population to ensure normal physical and biological activities; subsequently, social and economic activities are maintained. Many factors affect food security, including factors related to food production (agro-systems, innovations in food production, environmental factors, logistics, and international trade) and factors that affect overall economic growth (financial systems in place, fiscal and exchange rate policies), with the latter not directly controlled by food-production systems. Agro-systems, especially vertical indoor stations, underground horizontal farming, or hybrid horticulture plus fish-farming systems appear to be very promising; the underground system used by PlantLab (Netherland) was reported to produce three times the output of the best greenhouse system and 40 times that of open-field production [1]. Hydroponic systems appear to have even greater potential in terms of food production sustainability and food security. For example, production data reported from various systems indicated a range from 16 to 140 kg/m^2^ with a lower production input (90–95% less water use and less pesticides) and better opportunities to include smart technologies that reduce energy input [1]. The system appears to be versatile and can be adapted for food production on industrial rooftops and domestic residential areas. Urban agriculture is another opportunity to increase self-sufficiency and food security. Haberman et al. [2] predicted that the implementation of urban agriculture in vacant city spaces and residential gardens can boost vegetable production by >400%. More importantly, urban agriculture provides an excellent opportunity for direct access to fresh produce grown locally. 

Food innovations envisaged to ensure food security integrate novel (e.g., in vitro meat and milk production and bioprinted foods) and re-emerging (single-cell proteins, edible insects, fermentation, and enzyme technology) sources that enable the production of new food ingredients and the biotransformation of foodstuffs to reduce or remove antinutrients and undesirable food components. The latter technologies appear to offer the advantages of producing large amounts of food at a low production cost, in addition to the high nutritional quality of the generated foods.

## 3. Nutrition and Health Considerations in Food Innovation

One of the key United Nations Millennium Sustainable Development Goals (SDG) is the third SDG, which aims to promote the production of healthy food that delivers adequate nutrition and supports health and wellbeing. Current innovative food technologies place great emphasis on the sensory aspects of foods. For examples, most producers of alternative animal-based products focus on creating products that have similar sensory attributes to those of animal products. Additionally, the production of bioprinted products for senior citizens and special-needs consumers mostly focuses on acceptable sensory attributes and conferring health benefits. Recent innovations of in vitro, formulated, 3D-printed foods offer the opportunity to tailor the composition of foods through the supplementation of health-promoting nutrients or functional components that could improve certain targeted benefits (improve immune system, reduce oxidative stress, and so on). Nevertheless, the major change in food-production systems associated with future foods is yet to be fully appreciated and evaluated in terms of their safety and health impact. We do not have the same depth of understanding of novel foods that we do of conventional foods, and there are concerns over their safety due to the possible bioaccumulation of toxic contaminants, their microbiological safety, the deficiency of some essential amino acids, and protein digestibility [3]. There have been several reports of potential allergenicity in plants and insects [3,4], and with novel foods, new safety regulations are needed.

## 4. Public Health and Innovative Foods

Food safety and public health is global issue that affects millions of people around the world each year. According to the World Health Organization’s estimate, 600 million people (almost 1 in 10 people in the world) suffer from food poisoning after eating contaminated food, and 420,000 die every year [5]. There are more than 200 diseases (ranging from diarrhea to cancers) known to be caused by foods contaminated with harmful bacteria, viruses, parasites, or chemical substances. Innovative food products also expose consumers to known food-safety risks or new health risks due to the use of novel ingredients and/or emerging processing technologies. Furthermore, food-safety challenges are becoming complicated due to the multidimensional global food systems. The recent contamination of almond milk products with botulinum toxin provides a glimpse of the public health concerns that need to be managed for safe food supply in the future [6]. Food innovation, food safety, nutrition, and food security are inextricably linked and have significant impacts on the health of the public in many ways. 

This Special Issue aims to published quality articles on novel foods on a wide range of aspects including their role in food security, nutrition and health, and public health.
